# Optical coherence tomography-guided laser microsurgery for blood coagulation with continuous-wave laser diode

**DOI:** 10.1038/srep16739

**Published:** 2015-11-16

**Authors:** Feng-Yu Chang, Meng-Tsan Tsai, Zu-Yi Wang, Chun-Kai Chi, Cheng-Kuang Lee, Chih-Hsun Yang, Ming-Che Chan, Ya-Ju Lee

**Affiliations:** 1 Department of Electrical Engineering, Chang Gung University, 259, Wen-Hwa 1st Rd., Kwei-Shan Dist., Taoyuan city, 33302, Taiwan; 2 Medical Imaging Research Center, Institute for Radiological Research, Chang Gung University and Chang Gung Memorial Hospital at Linkou, Taoyuan, Taiwan; 3 Department of Dermatology, Chang Gung Memorial Hospital, 5 Fusing St., Kwei-Shan Dist., Taoyuan city, 33302, Taiwan; 4 Institute of Photonic System, College of Photonics, National Chiao Tung University, 301, Gaofa 3rd Rd., Guiren Dist., Tainan 71150, Taiwan; 5 Institute of Electro-Optical Science and Technology, National Taiwan Normal University, 88, Sec. 4, Ting-Chou Rd., Taipei 116, Taiwan

## Abstract

Blood coagulation is the clotting and subsequent dissolution of the clot following repair to the damaged tissue. However, inducing blood coagulation is difficult for some patients with homeostasis dysfunction or during surgery. In this study, we proposed a method to develop an integrated system that combines optical coherence tomography (OCT) and laser microsurgery for blood coagulation. Also, an algorithm for positioning of the treatment location from OCT images was developed. With OCT scanning, 2D/3D OCT images and angiography of tissue can be obtained simultaneously, enabling to noninvasively reconstruct the morphological and microvascular structures for real-time monitoring of changes in biological tissues during laser microsurgery. Instead of high-cost pulsed lasers, continuous-wave laser diodes (CW-LDs) with the central wavelengths of 450 nm and 532 nm are used for blood coagulation, corresponding to higher absorption coefficients of oxyhemoglobin and deoxyhemoglobin. Experimental results showed that the location of laser exposure can be accurately controlled with the proposed approach of imaging-based feedback positioning. Moreover, blood coagulation can be efficiently induced by CW-LDs and the coagulation process can be monitored in real-time with OCT. This technology enables to potentially provide accurate positioning for laser microsurgery and control the laser exposure to avoid extra damage by real-time OCT imaging.

Blood coagulation is a process where blood changes from a liquid state to a gel state in order to protect the damaged tissue and vessel following repair. However, for patients with blood coagulation disorders or undergoing surgery, dysfunctional coagulation causes bleeding or obstructive clotting^1^. Cryotherapy and radio frequency (RF) ablation are common solutions for therapy or vessel coagulation^2^,^3^,^4^,^5^,^6^. These treatment modalities induce a larger ablation area, resulting in extra damage to the surrounding tissue and difficulty with the treatment of specific areas or small vessels. Aside from tissue ablation, lasers have also been utilized to induce vascular occlusion and photocoagulation^7^,^8^,^9^. With laser exposure, the optical beam of treatment laser can be focused on a small area without causing extra damage to the surrounding tissue, making the treatment much safer and more effective. However, accurate determination of the treatment location and noninvasive monitoring of the treatment effect are important issues. To monitor the treatment effects and to improve the efficiencies of various treatment approaches, different imaging techniques has been intensively implemented such as magnetic resonance imaging (MRI)^10^,^11^,^12^, fluorescence microscopy^13^,^14^, confocal microscopy^15^,^16^, multiphoton microscopy^17^,^18^, laser speckle contrast imaging^19^,^20^, and optical coherence tomography^21^,^22^,^23^. However, identifying microstructures is difficult with MRI. In addition, microscopic techniques are difficult to probe the deeper structures and laser speckle contrast imaging is unable to provide depth-resolved information.

In contrast to the above techniques, optical coherence tomography (OCT) can provide high resolutions (<10 μm) and achieve a deeper imaging depth (<3 mm). Moreover, OCT is a noninvasive and label-free imaging technique^24^,^25^,^26^. In the past decade, the development of Fourier-domain OCT (FD-OCT) including spectral-domain OCT (SD-OCT)^27^,^28^,^29^, and swept-source OCT (SS-OCT)^30^,^31^,^32^ has realized high levels of sensitivity and imaging speed that are suitable for *in vivo* studies and diagnoses. In addition, different methods based on the OCT technique have been proposed for functional imaging to obtain various information on the angiography^33^,^34^,^35^,^36^,^37^, tissue birefringence^38^,^39^,^40^, and spectroscopic properties of tissue^41^,^42^.

Because of its high-speed imaging characteristic, OCT scanning can be used to observe the dynamic changes of biological tissues. This enables the real-time monitoring of morphological changes due to the interaction between photon energy and biological tissue. In this study, we propose a method to integrate laser microsurgery with OCT imaging for blood coagulation at small vessels or capillaries. Continuous-wave laser diodes (CW-LDs) in the visible spectral regime are used as a result of the higher absorption properties due to oxyhemoglobin and deoxyhemoglobin. Additionally, CW-LDs provide the advantage of cost-effectiveness over pulsed lasers for microsurgery. To accurately control the laser exposure location without damaging the surrounding tissue, we developed a method for image-based feedback positioning. Then, the blood coagulation process due to exposure to the CW-LDs can be observed from the OCT results. Furthermore, the cross-correlation coefficients of adjacent OCT images at the same location are estimated to observe the blood leakage and the coagulation process. Such an OCT-guided laser microsurgery system could be a powerful tool for various clinical applications such as dermatology, ophthalmology, and liver and brain surgery.

## Results

**Figure 1:**
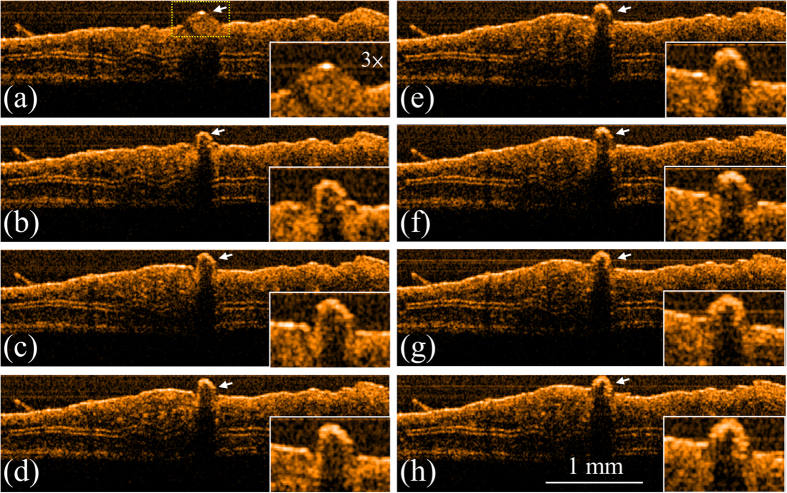
OCT results obtained for various exposure periods to 450-nm LD. (**a**) Before exposure; after exposure for (**b**) 3, (**c**) 6, (**d**) 9, (**e**) 12, (**f**) 15, (**g**) 18, and (**h**) 21 s. The ball shape indicated by the white arrow represents the blood leakage from the vessel caused by the needle. The region indicated by the yellow square in (**a**) is enlarged as shown in the right-lower corners.

**Figure 2:**
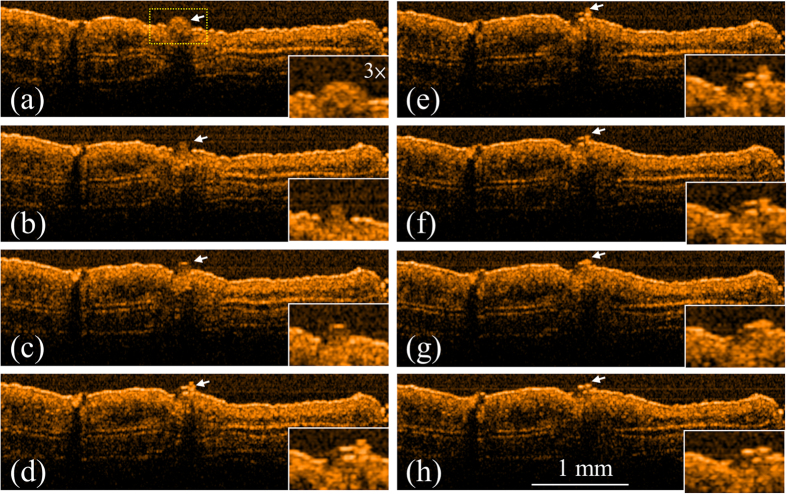
OCT results obtained for various exposure periods to 532-nm LD. (**a**) Before exposure; after exposure for (**b**) 3, (**c**) 6, (**d**) 9, (**e**) 12, (**f**) 15, (**g**) 18, and (**h**) 21 s. In (**a**), the white arrow indicates the region of leaked blood. The region indicated by the yellow square in (**a**) is enlarged as shown in the right-lower corners.

To evaluate the applicability of the integrated system to laser microsurgery, the ear skin of mice were experimented with the OCT-guided laser microsurgery system. To induce blood leakage, the vessel was punched with a needle before OCT scanning. Firstly, an LD with a center wavelength of 450 nm was used to induce blood coagulation. Figure 1 shows the OCT results obtained at various exposure periods: before laser exposure and after exposures for 3, 6, 9, 12, 15, 18, and 21 s. In Fig. 1(a), the ball shape indicated by the white arrow represents the blood leakage from the vessel, caused by the needle. To expose the specific area without damaging the surrounding tissue, the laser beam of the treatment LD was accurately positioned, according to the proposed method of image-based feedback positioning. During laser exposure, the optical beam of treatment LD was positioned at the center of blood drop and fixed without scanning to prevent from extra damage to surrounding tissue. From Fig. 1, it can be found that the leaked blood can be accurately exposed and the blood drop started to shrink after the laser exposure. Furthermore, the results of Fig. 1 showed that the blood started to coagulate after the laser exposure for 3 s. Then, the same experiment was repeated, and the other LD with a center wavelength of 532 nm was substituted for the 450-nm LD and used for laser treatment. Figure 2 shows the OCT images of the ear skin of the other mouse obtained before laser exposure and after the exposures for 3, 6, 9, 12, 15, 18, and 21 s, respectively. Similar to the results in Fig. 1, the blood started to coagulate after the laser exposure for 3 s. In addition, a blood clot can be observed after the laser exposure for 9 s, as shown in Fig. 2(d) and indicated by the white arrow. The results of Figs 1 and 2 showed that the areas indicated by the white arrow decreased as the exposure period increased.

**Figure 3:**
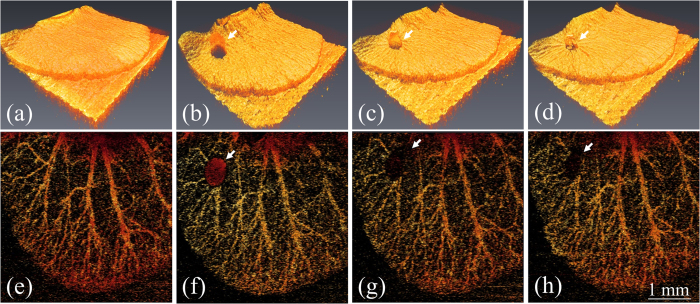
Top view of 3D OCT images and depth-encoded projection view of OCT angiography recorded (a,e) before blood leakage was induced by a needle, (b,f) during blood leakage, (c,g) after exposure to 532-nm laser for 5 s, and (d,h) after exposure to 532-nm laser for 10 s. The white arrows represent the region of leaked blood.

Moreover, to observe the blood leakage and obtain angiography in three dimensions, the ear skins of the mice were scanned with OCT to obtain 3D microstructural images, and the cross-correlation coefficients of the OCT images were estimated to acquire the angiography of the mouse ears and observe the blood leakage. Here, the moving particles, such as leaking blood cells from the vessels and moving blood cells in vessels, cause larger variations in backscattered intensities between two OCT images captured at the same location, resulting in low correlation. In contrast, the static particles or static tissue structures correspond to higher correlation between OCT images obtained at the same location. Therefore, the leaking blood cells and the moving blood cells can be visualized by extracting the regions of low correlation. To remove the contribution from the static particles, the regions of high correlation are rejected to acquire angiography and observe blood leakage. Again, the same procedures were repeated. Figure 3 shows the top view of the 3D OCT images and the depth-encoded projection view of the OCT angiography: (a, e) before blood leakage, (b, f) during blood leakage induced with a needle, (c, g) after exposure to the 532-nm laser for 5 s, and (d, h) after exposure to the 532-nm laser for 10 s. Before inducing blood leakage, no morphological change can be found in Fig. 3(a) and only angiography can be observed in Fig. 3(e). Then, the blood leaked from the vessel due to the punch of a needle. In Fig. 3(b), a ball shape indicated by the white arrow represents the leaking blood drop and then, the correlation coefficients were also estimated as shown in Fig. 3(f). In Fig. 3(f), the red spot indicated by the white arrow shows the leaking blood drop, corresponding to the lower correlation. Then, the laser beam of 532-nm laser was accurately controlled to focus on the leaking blood. After the 5-s laser exposure, the cross-correlation coefficients of the exposure area indicated by the white arrow in Fig. 3(g) became larger than those for Fig. 3(f). This was the result of the bleeding being suspended owing to blood coagulation. Moreover, the black area in Fig. 3(h) decreased when the exposure time was increased because of blood clotting. Additionally, it can be found from Fig. 3(d) that the skin surface became tightened. Therefore, the results showed that the OCT-guided laser microsurgery system enables accurate control of the treatment location to avoid extra damage and to monitor the treatment outcome with real-time coregistration.

## Discussion

**Figure 4:**
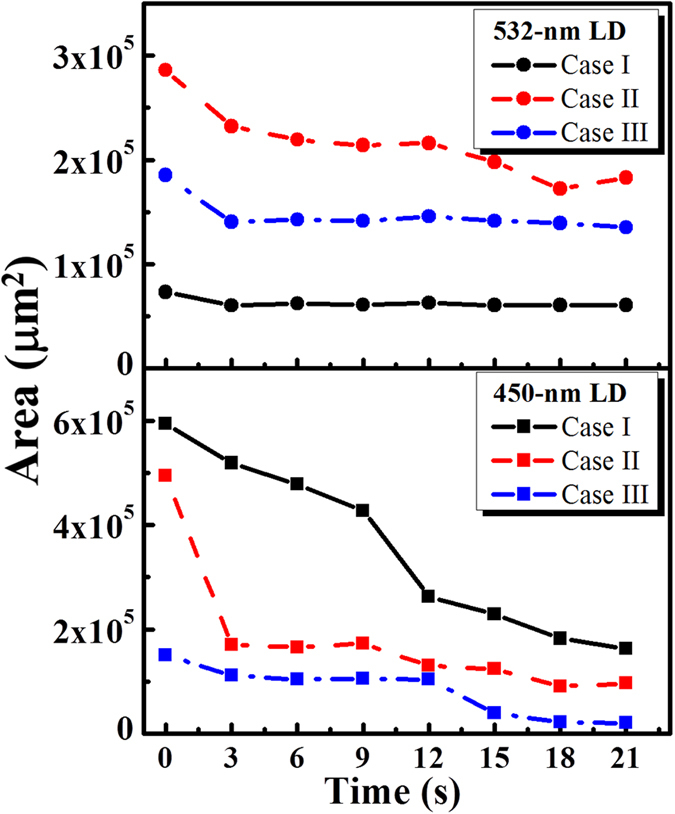
Estimated areas of the blood drops before and after laser exposures at various time points. The representative results from mice exposed with either a 450-nm LD (n = 3) or a 532-nm LD (n = 3) are shown . The lower and upper figures represent the estimated areas of the blood drops with 450-nm and 532-nm laser exposures for various periods.

Blood coagulation is a process to protect the damaged tissue and vessel following repair. However, for patients with blood coagulation disorders or undergoing surgery, dysfunctional coagulation causes bleeding or obstructive clotting. In this study, we develop an OCT-guided laser microsurgery system for blood coagulation with CW-LDs which can efficiently reduce the system cost. In addition, a method is proposed for accurate selection and positioning of the treatment location to avoid additional damage to the surrounding tissue. To quantitatively evaluate the morphological change as a function of exposure time, the areas of blood drops at various exposure periods were estimated, which was according to the proposed algorithm in the previous study^23^. Figure 4 shows the representative results from mice exposed with either a 450-nm LD (n = 3) or a 532-nm LD (n = 3). The lower and upper figures in Fig. 4 represent the estimated areas of the blood drops with 450-nm and 532-nm laser exposures for various exposure periods, which were estimated from the top-view images of 3D OCT results. The results showed that the areas of blood drops became smaller after laser exposure, indicating that leaked blood coagulated after the laser exposure for 3 s. As the exposure time was continuous to be increased, the areas of blood drops didn’t show a significant change. Based on the higher extinction coefficients resulting from the absorption of oxyhemoglobin and deoxyhemoglobin, two CW-LDs centered at 450 and 532 nm were used for experiments, and the results showed that both LDs can successfully induce blood coagulation. In comparison of the results of 450-nm LD and 532-nm LD exposures, the blood can be more efficiently coagulated after 450-nm LD exposure with the same exposure power, resulting from the higher extinction coefficient at 450 nm. However, the velocity of blood coagulation can be further improved by increasing the output power of CW-LDs. The results also showed that the treatment location can be precisely positioned and that the treatment outcome can be monitored with real-time coregistration. In the evaluation of the correlation coefficients, blood leakage and the suspension of bleeding were observed. The results showed that the proposed OCT-guided microsurgery system could be a powerful tool for various clinical applications such as dermatology, ophthalmology, and liver and brain surgery.

## Methods

**Figure 5:**
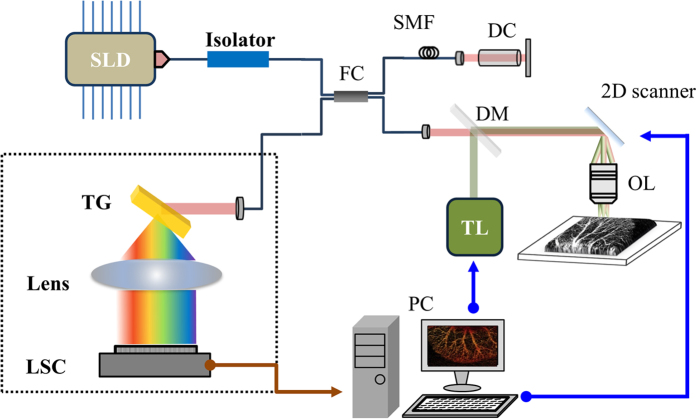
Schematic of setup for OCT-guided laser microsurgery. SLD: superluminescence diode; FC: fiber; SMF: single-mode fiber; DC: dispersion compensator; DM: dichroic mirror; TL: treatment laser; OL: objective lens; TG: transmission grating; LSC: linescan camera; PC: personal computer.

**Figure 6:**
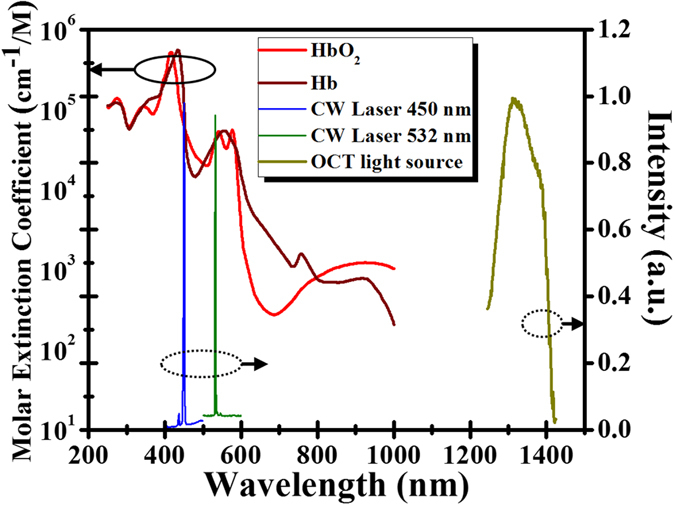
Spectra of two LDs for microsurgery, the OCT light source, and the absorption coefficients of oxyhemoglobin and deoxyhemoglobin 43.

Figure 5 shows a schematic of the integrated system combining laser microsurgery and the OCT system. To avoid unnecessary interference from the LD used for treatment, an SD-OCT system was developed for the 1.3-μm spectral regime. In the setup, a superluminescence diode (SLD) (SLD1325, Thorlabs Inc., NJ, USA) is connected to a fiber-based Michelson interferometer, and the light is split into the reference and sample arms. The center wavelength and full-width at half-maximum (FWHM) of the SLD are 1325 and 100 nm, respectively. The light source can provide an output power of more than 10 mW. In the sample arm, the OCT light beam is collimated and incident on a dichroic mirror (DM), which combines the laser beam for treatment and the OCT light beam. The two beams are incident on a 2D scanner and then focused by a 10× scanning lens (LSM02, Thorlabs Inc., NJ, USA). In addition, the dispersion induced by the scanning lens in the sample arm can be compensated by inserting a dispersion compensator in the reference arm. The fiber lengths of the two arms are accurately controlled to be equal to further minimize the dispersion effect. Finally, the interference intensity is detected with a spectrometer developed in-house; this consists of a collimator, transmission grating (1145 l/mm @ 1310) (Wasatch Photonics Inc., UT, USA), focus lens, and InGaAs linescan camera (LSC) (SUI1024LDH2, Sensors Unlimited Inc., NC, USA). In our experiments, the A-line rate of the LSC was set to 50,000 A-scan/s, which achieved a frame rate of 50 fps. A customized FPGA-based frame grabber was implemented for real-time display. Two CW-LDs are used to induce blood coagulation. The center wavelengths of the two LDs are 450 and 532 nm. Both LDs are driven with an output power of 2 W. Figure 6 shows the spectra of the two LDs, the OCT light source, and the molar extinction coefficients of oxyhemoglobin and deoxyhemoglobin^43^. From Fig. 6, two LDs for treatment correspond to higher extinction coefficients, enabling to maximize the efficiency of photocoagulation. In addition, because the 1.3-μm OCT system can probe the deeper tissue structure than that of an 800-nm OCT system, an SLD centered at 1.3 μm was chosen. Moreover, the SD-OCT system is used to prevent extra interference, originating from reflections of the treatment laser on the interfaces of optical components in the integrated system. In this setup, the interference induced by the treatment laser can be rejected by the spectrometer of our 1.3-μm OCT system. In the OCT system, the longitudinal and transverse resolutions in free space were measured to be approximately 8 and 7 μm, respectively. To obtain the vascular images from OCT images, the same B-scan location of tissue is scanned twice to obtain two OCT images. To obtain the 3D angiography, 500 B-scan locations are scanned. Thus, each 3D OCT volume comprises 1000 × 1000 × 512 voxels, which corresponds to a physical range of 4 mm × 4 mm × 2.5 mm. In our setup, the precision of laser positioning is limited to the focused spot size of treatment laser and the sampling intervals. In this case, the spot sizes of 405-nm and 532-nm LDs are 17 μm and 15 μm, respectively. In addition, the sampling intervals of the transverse and lateral directions are 4 μm and 8 μm, respectively.

To obtain the vascular image and observe blood leakage from the vessels, the estimation of cross-correlation coefficients, 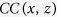, between two sequential OCT images at the same location can be expressed as


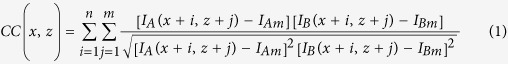


where *n* and *m* are the grid size. Here, both *n* and *m* equal 7. *I*_*A*_(*x*, *z*) and *I*_*B*_(*x*, *z*) indicate the OCT intensities of the two images^36^. Then, *I*_*Am*_ and *I*_*Bm*_ are the mean values of each grid. *x* and *z* represent the locations along the transverse and depth directions, respectively.

**Figure 7:**
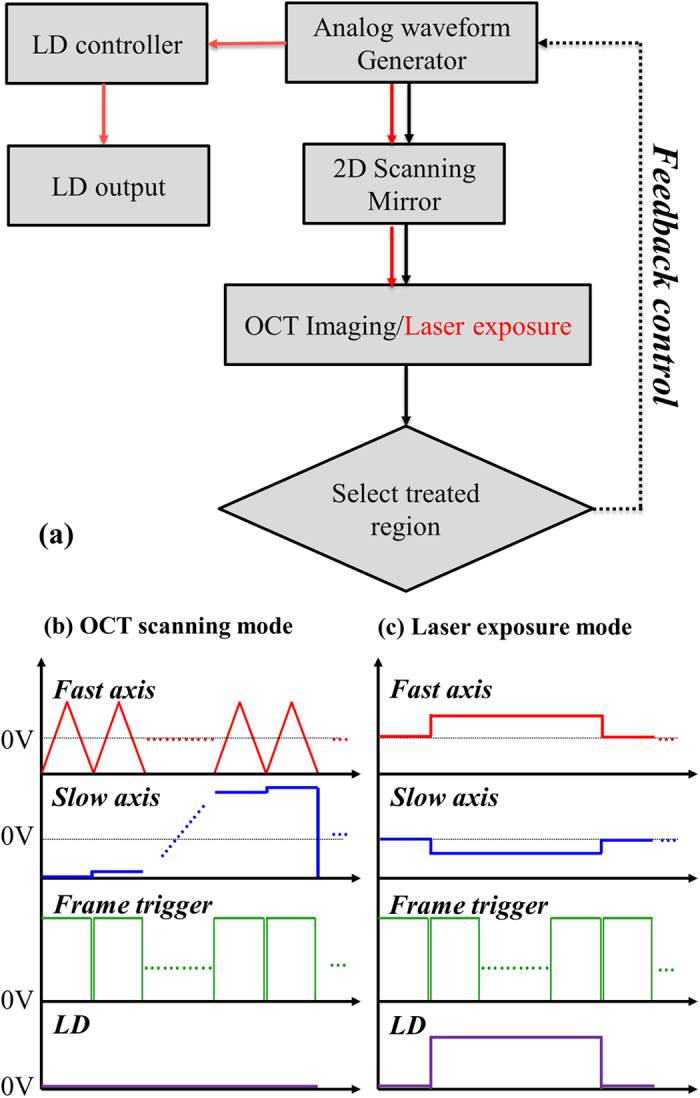
(**a**) Flowchart of image-based feedback positioning for laser microsurgery, (**b**) OCT scanning mode, and (**c**) laser exposure mode. The red and blue curves are used to drive the fast and slow axes, respectively, of 2D scanning. The green curves represent the waveform used to trigger image acquisition. The purple curve is used to control the exposure period of the LD.

In our experiment, mice (C57 wild-type, male, 7–8 weeks old) were anesthetized with a mixed anesthetic composed of oxygen and phenobarbital. The animal testing in this study was approved by the Laboratory Animal Center, Chang Gung University (IACUC Approval No: CGU13-126). In this study, a total amount of seventeen mice were included for 450-nm LD or 532-nm LD exposures. Eight mice were exposed to 450-nm LD and seven mice were exposed to 532-nm LD. The methods were carried out in accordance with the approved guidelines. To further reduce motion artifacts resulting from breathing, the mice were fixed on a specially designed mount fabricated by a 3D printer. Then, the mouse ears were scanned with the OCT system. To temporarily prevent blood self-coagulation, a low-dose anticoagulant was injected before OCT scanning. The vessel was punched with a needle to induce blood leakage. Subsequently, the leaked blood positioned by OCT images was exposed to the LD. In this experiment, we demonstrated a method for OCT image-based feedback control to accurately position the treatment location and noninvasively monitor the treatment outcome in real-time. Here, the area of blood drop can be identified based on the method demonstrated in the previous study and the center of blood drop can be determined^23^. Figure 7(a) shows a flowchart of image-based feedback positioning for laser microsurgery. A DAQ board (PXIe-6229, National Instruments Corp., Taiwan) with four channels for analog output is used to generate waveforms for driving the 2D scanner, synchronize the 2D scanner and LSC, and modulate the LDs to control the exposure period. In the OCT scanning mode, two waveforms including the triangular wave and step function are generated and connected to the drivers of the 2D scanner to provide beam scanning along the transverse and lateral directions, as shown in Fig. 7(b). Additionally, the LD is set to be off during the OCT scanning mode. Then, the exposure location (the center of blood drop) can be selected from 3D OCT images to determine the DC offset values for the two axes of the 2D scanner, as shown in Fig. 7(c). During laser exposure, the optical beam of treatment laser is fixed without scanning. The exposure period of the LD can also be adjusted by the controlled waveform, as indicated by the purple curve in Fig. 7(c). Moreover, M-mode OCT results can be recorded during laser exposure to enable real-time monitoring of the morphological changes at the treated location.

## Additional Information

**How to cite this article**: Chang, F.-Y. *et al.* Optical coherence tomography-guided laser microsurgery for blood coagulation with continuous-wave laser diode. *Sci. Rep.* **5**, 16739; doi: 10.1038/srep16739 (2015).
